# Manual for the Physiological Laboratory

**Published:** 1888-12

**Authors:** 


*Manual for the Physiological Laboratory.* By V. D. HARRIS, M.D. Lond., F.R.C.P., and D'ARCY POWER, M.A., M.B. Oxon., F.R.C.S. Fourth Edition. London: BALLIÈRE, TINDALL, and COX. 1888.

A book that has reached four editions obviously supplies a want, and supplies it satisfactorily, and therefore, like good wine, needs no bush. There are considerable improvements in the present edition; especially in the sections on Practical Physiology and Physiological Chemistry, the latter now occupying about twice the number of pages given to it in former editions. For the medical student this book supplies exactly the information he wants in a sound and reliable manner, and does not confuse him with details of experiments out of his compass. The practical directions are excellent; and among the minor advantages of the work are its clear type and neat binding.

